# Alteration of the blood-brain barrier by COVID-19 and its implication in the permeation of drugs into the brain

**DOI:** 10.3389/fncel.2023.1125109

**Published:** 2023-03-14

**Authors:** Héctor Hernández-Parra, Octavio Daniel Reyes-Hernández, Gabriela Figueroa-González, Manuel González-Del Carmen, Maykel González-Torres, Sheila I. Peña-Corona, Benjamín Florán, Hernán Cortés, Gerardo Leyva-Gómez

**Affiliations:** ^1^Departamento de Farmacología, Centro de Investigación y de Estudios Avanzados del Instituto Politécnico Nacional, Ciudad de México, Mexico; ^2^Departamento de Farmacia, Facultad de Química, Universidad Nacional Autónoma de México, Ciudad de México, Mexico; ^3^Laboratorio de Biología Molecular del Cáncer, UMIEZ, Facultad de Estudios Superiores Zaragoza, Universidad Nacional Autónoma de México, Ciudad de México, Mexico; ^4^Laboratorio de Farmacogenética, UMIEZ, Facultad de Estudios Superiores Zaragoza, Universidad Nacional Autónoma de México, Ciudad de México, Mexico; ^5^Facultad de Medicina, Universidad Veracruzana, Heroica Veracruz, Mexico; ^6^Conacyt and Laboratorio de Biotecnología, Instituto Nacional de Rehabilitación “Luís Guillermo Ibarra”, Ciudad de Mexico, Mexico; ^7^Departamento de Fisiología, Biofísica y Neurociencias, Centro de Investigación y de Estudios Avanzados del Instituto Politécnico Nacional, Mexico City, Mexico; ^8^Laboratorio de Medicina Genómica, Departamento de Genómica, Instituto Nacional de Rehabilitación Luis Guillermo Ibarra Ibarra, Ciudad de México, Mexico

**Keywords:** blood-brain barrier, drug permeation, central nervous system, COVID-19, immune response

## Abstract

Diverse neurological symptoms have been reported in patients with SARS-CoV-2 disease (COVID-19), including stroke, ataxia, meningitis, encephalitis, and cognitive impairment. These alterations can cause serious sequelae or death and are associated with the entry of SARS-CoV-2 into the Central Nervous System (CNS). This mini-review discusses the main proposed mechanisms by which SARS-CoV-2 interacts with the blood-brain barrier (BBB) and its involvement in the passage of drugs into the CNS. We performed a search in PubMed with the terms “COVID-19” or “SARS-CoV-2” and “blood-brain barrier injury” or “brain injury” from the year 2019 to 2022. We found proposed evidence that SARS-CoV-2 infects neurovascular cells and increases BBB permeability by increasing the expression of matrix metalloproteinase-9 that degrades type IV collagen in the basement membrane and through activating RhoA, which induces restructuring of the cytoskeleton and alters the integrity of the barrier. The breakdown of the BBB triggers a severe inflammatory response, causing the cytokine storm (release of IL-1β, IL-6, TNF-α, etc.) characteristic of the severe phase of COVID-19, which includes the recruitment of macrophages and lymphocytes and the activation of astrocytes and microglia. We conclude that the increased permeability of the BBB would allow the passage of drugs that would not reach the brain in a normal physiological state, thus enhancing certain drugs’ beneficial or adverse effects. We hope this article will encourage research on the impact of drugs on patients with COVID-19 and recovered patients with sequelae, focusing mainly on possible dose adjustments and changes in pharmacokinetic parameters.

## 1. Introduction

Severe acute respiratory syndrome coronavirus 2 (SARS-CoV-2) is the cause of coronavirus disease 2019 (COVID-19), which has caused more than 6.5 million deaths and more than 621 million positive cases worldwide (at least until October 17, 2022) as reported by the World Health Organization [WHO] (2022). According to a report by Visual Capitalist (with data from the WHO), COVID-19 is the pandemic with the seventh-highest number of deaths in modern history (Visual Capitalist, 2020).

SARS-CoV-2 is a positive-sense, single-stranded RNA virus with a spherical and spiked protein envelope of about 60-140 nm, belonging to the betacoronavirus genus (Welcome and Mastorakis, 2021). Among the main structural proteins, there is the spike (S) glycoprotein, which is a type I transmembrane protein, composed of the S1 subunit responsible for binding to the receptor on the surface of the host cell and the S2 subunit responsible for the fusion of membranes and viral penetration (Tizenberg et al., 2021; Takeda, 2022). SARS-CoV-1 and SARS-CoV-2 share 79% identity with each other (Lu et al., 2020; Chen Z. et al., 2021); however, SARS-CoV-2 has shown higher binding affinity to human angiotensin-converting enzyme 2 (ACE2), which has been identified as the primary mechanism of cellular infection (Tizenberg et al., 2021). Therefore, SARS-CoV-2 affects the lungs, kidneys, heart, liver, pharynx, brain, and all organs exhibiting ACE2 receptors expression (Puelles et al., 2020). Several neurological complications have been reported in COVID-19 patients, including seizures, Guillain-Barré syndrome, encephalitis, dizziness, headache, ageusia, anosmia, cognitive impairment, affective disorders, coordination deficit, and cerebrovascular injury (Asadi-Pooya and Simani, 2020; Berlit et al., 2020; Mao et al., 2020; Ermis et al., 2021; Papri et al., 2021). In this regard, brain involvement could occur through direct damage to the blood-brain barrier (BBB) that leads to the permeation and spread of the virus into the central nervous system (CNS) (Krasemann et al., 2022). It should be noted that there are other ways the virus can enter the brain, such as the olfactory nerve pathway, where SARS-CoV-2 binds to the olfactory bulb and sustentacular cells. Previous studies have shown that several viruses can enter the brain through this pathway, including SARS-CoV-1, MERS-CoV, and HCoV-OCR43 (Wang et al., 2020). The theory that SARS-CoV-2 can enter through the gastrointestinal system to invade the enteric nervous system and, finally, the brain has also been proposed (Wu and Tang, 2020). However, this review focuses on the BBB as the primary physical defense that regulates the transport of drugs and other substances to the brain.

The BBB is a complex structure of endothelial cells (ECs) regulated by pericytes and end-feet of the astrocytes, as well as vascular smooth muscle cells that contribute to the integrity of the BBB, which dynamically control permeability and prevent the entry of harmful agents and pathogens into the brain. The ECs are connected by tight junctions (TJs) that limit the movement of substances through the paracellular space. Nonetheless, small lipophilic molecules with molecular weight <600 Da may diffuse across the BBB to enter the brain (Zhou et al., 2018). For greater effectiveness in drug delivery, temporary interruption of the BBB has been tried by various methods (Chen et al., 2022). However, this strategy has high risks, such as the invasion by immune cells, bacteria, viruses, toxins, or other molecules (including drugs) that may cause unwanted effects on the brain.

## 2. Immune response to SARS-CoV-2 and cytokine storm

Once the SARS-CoV-2 infection begins, the virus replicates and is transmitted to adjacent cells, generating cellular signals that activate innate sentinel cells such as macrophages, mast cells, dendritic cells, natural killer cells, and other defensive mechanisms such as the complement system (Sanz et al., 2021). Macrophages can be activated by the virus or infected cells, releasing proinflammatory cytokines that affect cell permeability and composition (Sanz et al., 2021). Professional antigen-presenting cells, such as dendritic cells, pick up viral particles, mature, and migrate to secondary lymphoid organs. At these sites, it takes place the presentation of viral peptides and the activation of virus-specific T helper (Th) and cytotoxic (Tc) lymphocytes, which proliferate and release large amounts of cytokines, leading to the lysis of infected cells (Jansen et al., 2019). Under normal conditions, this immune system response can stop the infection and generate cellular antigenic memory. However, SARS-CoV-2 can alter the immune response and aggravate the disease (Sanz et al., 2021). Direct CNS infection and systemic inflammation compromise the BBB and trigger a massive neuroinflammatory manifested by reactive astrogliosis and microglia activation (Steardo et al., 2020). Activated microglial cells induce the release of cytokines, which further activate astrocytes. Activated astrocytes release mediators, such as TNF, prostaglandins, and glutamate, which are involved in neurotoxicity induced by neuroinflammation (Almutairi et al., 2021).

The cytokine storm is a widely studied phenomenon in COVID-19 and has been associated with severe manifestations of the disease; it is a state of hyperinflammation, often irreversible and fatal (Davidson et al., 2015; Guiomar-Córdova et al., 2021; Chen et al., 2022). The release of primary proinflammatory cytokines, such as IFN-γ and TNF-α, lead to the activation of immune cells, such as macrophages, dendritic cells, neutrophils, T cells, B cells, mast cells, and EC, which secrete cytokines, including IL-2, IL-6, IL-7, IL-8, IL-1β, and granulocyte-macrophage colony-stimulating factor (Zirpe et al., 2020; Sanz et al., 2021). Some authors have reported that IL-6 further activates immune cells, which can lead to excessive activation of the complement system and coagulation cascades that contribute to a harmful cycle of cytokine storm (Han et al., 2021). It has also been reported that upon microglia activation, they release molecular signals such as IL-1 and TNF-α, which activate astrocytes. Activated astrocytes produce inflammatory factors, including TNF-α, ROS, and nitric oxide (NO), in response to microglial activation. This communication between microglia and astrocytes amplifies the neuroinflammation cascade (Almutairi et al., 2021). A large number of cytokines in a short time and overactivation of immune cells leads to severe disease manifestations or even death (Shimabukuro-Vornhagen et al., 2018; Chen et al., 2022). This secondary inflammatory reaction destabilizes the tight junctions and damages the ECs and astrocytes of the BBB, increasing vascular permeability and facilitating viral entry (see section “5. Mechanisms involved in the permeation of the BBB by SARS-CoV-2”) (Chen et al., 2022).

## 3. Neurological manifestations

Neurological symptoms have been reported frequently in patients with severe COVID-19. During the first months of the pandemic outbreak in Mao et al. (2020) investigated a cohort of 214 patients positive for COVID-19, from which 36.4% had neurological complications, mainly those with severe infection. The neurological complications associated with SARS-CoV-2 include encephalitis, meningitis, dizziness, seizures, ageusia, anosmia, altered consciousness, affective disorders, coordination deficit, paresis, ataxia, acute disseminated encephalomyelitis, cerebrovascular injury, and cognitive impairment (Kumar et al., 2020; Mao et al., 2020; Ermis et al., 2021). A cross-sectional clinical study that included 35 patients who survived COVID-19 and had no medical history of dementia or cognitive impairment found that patients with the disease were at risk of developing some cognitive impairment after recovery (Siddig et al., 2022). Other studies have also reported long-term neurological manifestations termed “Long Covid,” including concentration problems, headache, sensory disturbances, and depression, which can persist for months after infection (Burks et al., 2021; Spudich and Nath, 2022). For example, some cases of parkinsonism have been linked to SARS-CoV-2 infection, raising the possibility of a post-viral parkinsonian syndrome (Cavallieri et al., 2022). A possible explanation is that the cytokine storm caused by the infection may produce minor punctual cerebrovascular accidents without causing significant neurological deficits, which may worsen after overcoming the disease and have been associated with cognitive and attention problems (Guiomar-Córdova et al., 2021). On the other hand, and in this same context, there is the possibility that SARS-CoV-2 persists in resident cells of the CNS and may be related to the development of long-term neurological manifestations. However, it is an idea that still needs to be explored.

## 4. Detection of SARS-CoV-2 in the central nervous system and neuroinvasive potential

There is strong evidence that SARS-CoV-2 can infect the CNS because it has been detected in cerebrospinal fluid from living patients and post-mortem brain tissue from patients with COVID-19 (Xu et al., 2005; Baig et al., 2020; Tizenberg et al., 2021; Krasemann et al., 2022). In contrast, Lee et al. (2022) failed to detect viral particles in the brain during an autopsy study of deceased COVID-19 patients but did find brain regions with signs of inflammation. Interestingly, the patients included in this study died after a short infection duration, and several died suddenly with minimal respiratory involvement (Lee et al., 2022). There is a possibility that the virus has not yet been able to enter the CNS at the time of death, and the alterations in brain regions are likely due to peripheral inflammatory processes. Peripheral proinflammatory cytokines can induce cytokine synthesis within the CNS (Ferrari, 2021). Therefore, there may be cases in which the virus affects the brain, neurons, and glia before it can enter this site. However, the virus can exacerbate all this damage once it enters the brain.

On the other hand, Zhang et al. (2021) used fluorescence *in situ* hybridization, transmission electron microscopy, and immunostaining to demonstrate that SARS-CoV-2 infects brain ECs in animal models (mice and hamsters). Likewise, Song et al. (2021) examined formalin-fixed, paraffin-embedded brain sections from three patients who died after severe complications related to COVID-19. They identified anti-spike antibody staining in cortical neurons and ECs (Song et al., 2021). This evidence was consistent with another study that detected the virus in cortical neurons in autopsies of patients with COVID-19 and demonstrated ECs susceptibility by infecting human-induced pluripotent stem cell-derived brain capillary endothelial-like cells (Krasemann et al., 2022). Finally, Crunfli et al. (2022) performed brain autopsies on 26 people who died from COVID-19 and sampled five brains with histopathologic abnormalities. They detected genetic material and protein S from SARS-CoV-2 in all five samples, indicating neuroinvasion. Specifically, they identified protein S in 37% of brain tissue cells, where most of these spike-positive cells (65.93%) were astrocytes. They also reported that *in vitro* neural stem cell-derived human astrocytes are susceptible to SARS-CoV-2 infection through interaction with the Neuropilin-1 receptor (NRP1) because the NRP1 blockade with neutralizing antibodies reduces SARS-CoV-2 infection considerably (Crunfli et al., 2022). Therefore, although more evidence is needed to determine which are the most susceptible brain cells, the invasive capacity of SARS-CoV-2 has been demonstrated in the key cells that control the permeability of the BBB: ECs and astrocytes.

## 5. Mechanisms involved in the permeation of the BBB by SARS-CoV-2

**FIGURE 1:**
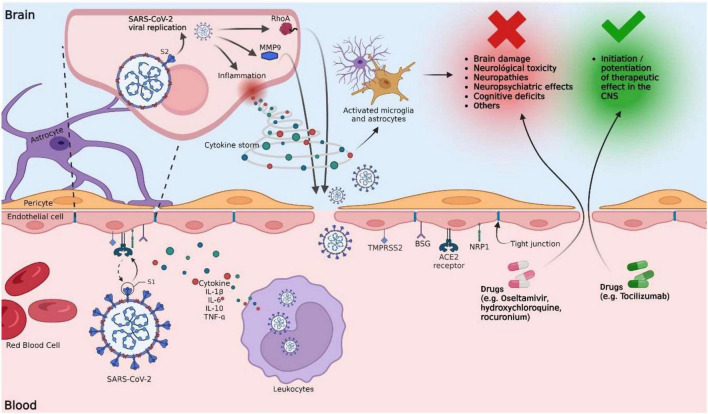
Potential mechanisms of the blood-brain barrier disruption by SARS-CoV-2 and their implication in the increased permeability of drugs into the brain. SARS-CoV-2 interacts with the BBB by binding the viral S protein and the ACE2 receptors of the vascular endothelium. Other proteins, such as NRP1, TMPRSS2, BSG, and Cathepsin L, are also involved. Proteolytic activation of the S protein is a crucial step in SARS-CoV-2 infection and occurs at the S1/S2 site by the action of furin protease. After binding to ACE2, the S protein undergoes further cleavage mediated by TMPRSS2 at the S2’ site to facilitate the fusion of the virus with the host cell membrane. Once the infection of BBB ECs has occurred, viral replication continues, promoting the activation of inflammatory mechanisms, which include the release of proinflammatory cytokines. The release of primary proinflammatory cytokines, such as IFN-γ and TNF-α, leads to the activation of immune cells, ECs, astrocytes, and microglia. Activated microglial cells induce the release of cytokines such as IL-1, IL-6, and TNF-α, which further activate astrocytes. Activated astrocytes release mediators, such as TNF, prostaglandins, glutamate, ROS, and nitric oxide, which are implicated in neurotoxicity induced by neuroinflammation. IL-6 can cause excessive activation of the complement system and coagulation cascades that contribute to a harmful cytokine storm cycle, destabilizing TJs and damaging BBB ECs, increasing vascular permeability. Lysis of infected ECs alters the permeability of the BBB. The virus promotes an increase in the expression of MMP9 (responsible for the degradation of collagen IV, an essential component of the basal membrane), promoting the rupture of the BBB. S protein promotes RhoA activation, which induces cytoskeletal restructuring and TJs disassembly, altering BBB permeability. Following these possible mechanisms, the BBB is exposed to the passage of xenobiotics, including viral particles and drugs. Drugs such as oseltamivir have limited penetration through the BBB because it is a substrate for P-glycoprotein. However, in a clinical setting, BBB disruption could increase oseltamivir penetration into the brain, potentially leading to increased unwanted CNS effects. On the contrary, tocilizumab has a limited ability to cross the BBB; however, its therapeutic effect would be enhanced with a more permeable BBB.

Although elucidating the mechanisms involved in the invasion of SARS-CoV-2 into the CNS remains a challenge, it is known that this virus can enter the brain through the olfactory and hematogenous pathways (Burks et al., 2021; Han et al., 2021). The neuroinvasion mechanism through the olfactory pathway is supported by brain magnetic resonance (MRI) studies of people with COVID-19, where structural changes have been found in the olfactory nerve, the olfactory bulb, and the cerebral cortex (Tizenberg et al., 2021). The hematogenous route suggests that the virus can spread in the bloodstream and reach the brain through the BBB, so here we focus on possible mechanisms that alter the permeability of this barrier (Figure 1). The primary invasion mechanism of SARS-CoV-2 toward the BBB is binding between the viral S protein and the ACE2 receptors found in the vascular endothelium. However, they have also been found in smooth muscle, neurons, and glia (Hamming et al., 2004; Chen R. et al., 2021). Moreover, other crucial proteins for the infection and spread of SARS-CoV-2 in the CNS have also been identified, including NRP1, transmembrane protease serine 2 (TMPRSS2), basigin, and cathepsin L (Burks et al., 2021; Song et al., 2021; Welcome and Mastorakis, 2021). The S protein has two cleavage sites (S1/S2 and S2’). Proteolytic activation of protein S is a crucial step in SARS-CoV-2 infection, and it occurs at the S1/S2 site by the action of furin protease (Takeda, 2022). After binding to ACE2, the S protein undergoes additional cleavage at the S2’ site, mediated by TMPRSS2, to facilitate the fusion of the virus with the host cell membrane (Kumar et al., 2020; Takeda, 2022). When the virus infects the BBB’s ECs, it continues its viral replication, promoting neuroglial inflammation (Welcome and Mastorakis, 2021). In addition, the release of inflammatory cytokines in response to viral infection alters the permeability of the BBB, which could lead to a higher degree of CNS infection by allowing easy entry of viral particles (Burks et al., 2021; Han et al., 2021).

A brain autopsy study assessed BBB integrity in patients deceased by COVID-19 (Lee et al., 2022). Immunostaining for fibrinogen showed areas of multifocal staining throughout the brain, reporting platelet aggregates and microthrombi adhering to ECs throughout the vascular lumen, which suggests BBB lesions that allow leakage of serum proteins into the brain parenchyma. To elucidate the possible mechanisms of BBB rupture, Zhang et al. (2021) administered Evans blue dye in hamsters infected with SARS-CoV-2. They found the destruction of basement membranes in the cortices without alteration in the expression of claudin-5, zonula occludens-1 (ZO-1), occludin, and the TJs ultrastructure. Moreover, they identified an increase in the expression of matrix metalloproteinase-9 (MMP9), which could explain the degradation of collagen IV, the destruction of the basement membrane, and the rupture of the BBB.

On the other hand, DeOre et al. (2021) reported that protein S could also alter the BBB by activating RhoA, which induces cytoskeleton restructuring and disrupts the barrier by TJs disassembly. Inhibition of RhoA with C3 transferase in the presence of protein S rescued prevented these effects. Likewise, immunofluorescence assays verified that RhoA inhibition rescued ZO-1 compartmentalization in TJs in the presence of protein S.

TJs between adjacent endothelial cells form the basic structure of the BBB and play a central role in the paracellular trafficking of the virus. During infection, SARS-CoV-2 can infect ECs and cross the BBB through two pathways (1) by a transcellular pathway through MMP9-mediated disruption of the basement membrane and (2) a paracellular pathway through RhoA activation and TJs disassembly. However, more exhaustive studies are required to determine under which condition MMP9 overexpression occurs, which seems to increase BBB permeability without altering TJs, which activates RhoA, and which condition promotes increased permeability by altering TJs.

## 6. Modification in drug transport through the BBB altered by SARS-CoV-2

The rupture of the BBB would allow the passage of unwanted substances into the CNS, including drugs that are not typically permeable or of no benefit to the CNS. Instead, it might produce toxic effects that could be severe or even fatal. Antivirals such as remdesivir, lopinavir, ritonavir, and oseltamivir have been included in the therapeutic recommendations of the National Institutes of Health to treat COVID-19 (National Institutes of Health [NIH], 2022). Nevertheless, in some cases, specific antiviral therapy has been associated with neurological toxicity, characterized by peripheral neuropathy and neurocognitive and neuropsychiatric effects. For example, oseltamivir has been documented to be related to neurological toxicity (Ono et al., 2018). A large-scale analysis of data from the US Food and Drug Administration Adverse Event Reporting System reported neuropsychiatric adverse events associated with treatment with oseltamivir in cases of SARS-CoV-2 infection, which were described as abnormal behavior, hallucinations, and seizures (Punekar et al., 2022). Oseltamivir has limited penetration through the BBB because it is a P-glycoprotein substrate (DrugBank, 2022). However, in a clinical setting, disruption of the BBB could increase the permeation of oseltamivir (and other drugs) into the brain, leading to harmful effects or worsening of known CNS effects.

On the other hand, neuromuscular blocking agents, such as rocuronium, are used for the treatment of patients with acute respiratory distress syndrome (ARDS), moderate and severe, and have not been reported to have adverse CNS reactions (Zakynthinos et al., 2021). Moreover, it has also been described that rocuronium cannot cross the BBB due to its limited liposolubility (Jain et al., 2022). However, in a case series, three patients with ARDS due to COVID-19 and treated with rocuronium presented bilateral non-reactive dilated pupils. Discontinuation of rocuronium led to the reversal of pupillary dilation. Therefore, it is possible that the alteration of the BBB by SARS-CoV-2 allowed the drug access to the CNS and caused this adverse effect (Zakynthinos et al., 2021). Interestingly, re-exposure to rocuronium in one patient caused a recurrence of pupillary dilation, despite the patient’s negative PCR for SARS-CoV-2. This finding could indicate that BBB damage might last for a prolonged period after overcoming the infection and could help explain the reports of Long Covid sequelae (Zakynthinos et al., 2021).

Finally, tocilizumab is an anti-IL-6 receptor used in the treatment of COVID-19. According to the National Institutes of Health COVID-19 treatment guidelines, tocilizumab is recommended for critically ill hospitalized patients who require high-flow oxygen or more intensive respiratory support. Patients treated with tocilizumab infusion have been reported to survive longer than patients who did not, and this correlated with serum IL-6 levels (Quartuccio et al., 2020). Large randomized clinical trials such as REMAP-CAP, RECOVERY, and PROSPERO showed improved survival rates and clinical outcomes for patients treated with IL-6 receptor blockers (Schultz et al., 2023). Tocilizumab has a limited ability to cross the BBB (Richardson et al., 2020); however, when patients have a more permeable BBB, this drug can pass freely and reach the CNS microenvironment in pathological conditions. Therefore, contrary to other medications, disruption of BBB would improve the therapeutic effects of tocilizumab (Schultz et al., 2023).

Advanced MRI techniques could be used to elucidate in more detail the possible alteration in the permeability of the BBB, such as the analysis of contrast leaks that allows assessing the state of BBB permeability. Damage levels in the BBB could also be analyzed using a convolutional neural network (CNN) to segment MR brain images (el Kader et al., 2022). These types of studies can provide timely information on the status of the BBB and could support physicians’ decisions during drug treatment.

## 7. Conclusion

The main mechanisms involved in the interruption of the BBB that could be responsible for the severe neurological symptoms in COVID-19 are: (1) hyperinflammation by infection of ECs and surrounding immune cells, which triggers an inflammatory cytokine storm that would lead to the lysis of infected cells; (2) upregulated expression of matrix metalloproteinase-9, which degrades collagen IV, an essential component for basement membrane stability; and (3) activation of RhoA, a small GTPase that induces cytoskeleton restructuring and TJs disassembly. These mechanisms compromise the integrity of the BBB and allow the extravasation of leukocytes, the leakage of plasmatic proinflammatory agents such as cytokines, the passage of circulating SARS-CoV-2 particles toward the brain parenchyma, and the free passage of drugs. In this article, we propose an integrative mechanism to explain the possible alteration in the BBB permeability by COVID-19 and its implication in pharmacological treatment. We suggest that it is crucial to consider the possibility of increased BBB permeability when prescribing medications for COVID-19 since it turns out to be a double-edged sword. On the one hand, it could allow for the cerebral effect of drugs that generally cannot reach the brain; on the other hand, it could increase the toxic effects of certain medications.

## Author contributions

HH-P and GL-G conceptualized the work. HH-P, OR-H, GF-G, MG-DC, MG-T, SP-C, BF, and HC wrote the draft manuscript. HH-P and SP-C created the Figure 1. BF, HC, and GL-G supervised and edited the final version of the manuscript and obtained the funding. All authors contributed to the final version of the manuscript and approved the submitted version.
